# Biosafety Evaluation of 6,7‐Dihydroxy‐3‐(2‐Nitrophenyl)Coumarin in Human Cells

**DOI:** 10.1002/jat.4880

**Published:** 2025-08-05

**Authors:** Júlia Zocca Nunes de Barros, Luma Eduarda Lopes Escalante, Maria João Matos, Edson Luis Maistro

**Affiliations:** ^1^ Faculdade de Medicina de Marilia – FAMEMA Marília Brazil; ^2^ Speech and Hearing Therapy Department, Faculty of Philosophy and Sciences São Paulo State University (UNESP) Marília SP Brazil; ^3^ Departamento de Química Orgánica, Facultade de Farmacia Universidade de Santiago de Compostela Santiago de Compostela Spain

**Keywords:** 6,7‐dihydroxy‐3‐(2‐nitrophenyl)coumarin, comet assay, HepG2/C3A cells, micronucleus assay, Perkin–Oglialoro reaction, resazurin assay

## Abstract

Coumarins are considered a privileged scaffold in medicinal chemistry, as they may interact with biological macromolecules, presenting several pharmacological properties. Their potential makes them an object of study for the evaluation of their safety for humans, which is essential in the design of a potential drug. 6,7‐Dihydroxy‐3‐(2‐nitrophenyl)coumarin is a synthetic derivative with antioxidant properties, amongst other biological activities under study. The main goal of this study is to evaluate the potential cytotoxic and genotoxic effects of this molecule in peripheral blood mononuclear cells (PBMCs) and human hepatocellular carcinoma cells (HepG2/C3A). The results obtained for the cytotoxicity assays, evaluated by the resazurin assay, using concentrations between 0.1 and 50 μg/mL, showed that there is no decrease in cell viability for both cell lines. Regarding genotoxic assays, the data obtained by the comet assay and the micronucleus test, up to a concentration of 30 μg/mL, did not show significant DNA damage and/or chromosomal mutations for both cell types. Given these results, it can be concluded that 6,7‐dihydroxy‐3‐(2‐nitrophenyl)coumarin, up to a concentration of 30 μg/mL, does not present cytotoxic or genotoxic effects in human cells with and without hepatic metabolism. Considering that this family of coumarins, in general, presents their biological effect at low concentrations (mainly nanomolar range), the results obtained here encourage further studies with this molecule in drug discovery programs. 6,7‐Dihydroxy‐3‐(2‐nitrophenyl)coumarin is a new synthetic derivative with antioxidant properties, amongst other biological activities under study. The main goal of this study was to evaluate the potential cytotoxicity and genotoxicity of this molecule in human cells in vitro. Cytotoxicity assays in concentrations between 0.1 and 50 μg/mL showed no decrease in cell viability. Comet assay and the micronucleus test, up to a concentration of 30 μg/mL, did not show genetic toxicity.

## Abstract 2

6,7‐Dihydroxy‐3‐(2‐nitrophenyl)coumarin is a new synthetic derivative with antioxidant properties, among other biological activities under study. The main goal of this study was to evaluate the potential cytotoxicity and genotoxicity of this molecule in human cells in vitro. Cytotoxicity assays at concentrations between 0.1 and 50 μg/mL showed no decrease in cell viability. The comet assay and the micronucleus test, up to a concentration of 30 μg/mL, did not show genetic toxicity.

## 1 Introduction

Coumarins, initially isolated by Vogel in 1820 from *Dipterys odorata*, known as cumaru, are considered a derivative of benzoic acid, widely distributed in the plant kingdom, particularly plants belonging to the natural orders of *Orchidaceae*, *Leguminoceae*, *Rutaceae*, *Umbelliferae*, and *Labiatae* (Sethna and Shah 1945; Franco et al. 2020). Through hydrolysis, coumarins provide one or more sugars to living organisms, releasing secondary metabolites. They can naturally be present in the roots, flowers, and fruits of guaco, cinnamon, watercress, cherry, strawberry, and apricot. The relationship between coumarin and *ortho*‐hydroxycinnamic acid appeared from the synthesis described by Perkin, which occurs through shikimic acid, which is not restricted to plants and can be found in some species of fungi and bacteria (Franco et al. 2020; Johnson 2011; Dighe et al. 2010). This and other methodologies allowed the family of synthetic coumarins to grow.

Structurally, a coumarin is formed by a pyrone ring fused to benzene, with the carbonyl at position 2 (Dighe et al. 2010). This structure gives the molecule the ability to exert non‐covalent interactions with proteins, explaining why coumarins have been widely investigated as bioactive compounds. They present a variety of biological properties depending on the substitution patterns, contributing to cutting‐edge research looking for new pharmacological properties, as well as enabling the research for innovative synthetic routes to obtain new derivatives (Garrett 2010). Coumarins may display antiviral, antimicrobial, anti‐inflammatory, anticoagulant, anticancer, anticonvulsant, antineurodegenerative, and analgesic properties (Wu et al. 2009; Junior et al. 2015; Matos et al. 2021, 2022, 2023).

Treatment with medicinal plants is widely used for health recovery and prevention. Phytotherapy is integrated into the culture of specific populations, being passed down through generations over decades and centuries, increasing scientific evidence of the use of medicinal plants (Soares et al. 2021). On the other hand, some plants have the ability to produce different toxic substances in different amounts, as a form of defense against microorganisms and predatory animals. For this reason, toxicologic and genotoxic studies involving extracts and isolated molecules are of great relevance, considering the recognized interrelationship between mutagenicity and carcinogenicity (Dearfield and Moore 2005; Gomes et al. 2017). Considering that a variety of compounds are capable of damaging the DNA of living cells, and that genotoxic potential can lead to lethality, hereditary effects, and cancer, cytotoxicity and genotoxicity tests are essential to identify the safety profile of any compound, allowing knowledge of their risk to living beings and the environment (Valente et al. 2017; Oliveira 2018; Almeida 2021). Therefore, the main goal of this study is to investigate the cytogenotoxic potential of 6,7‐dihydroxy‐3‐(2‐nitrophenyl)coumarin in human cells with and without the capacity for hepatic metabolization.

## 2 Material and Methods

### 2.1 Chemistry

#### 2.1.1 General Information

All reagents were purchased from Merck and used without further purification. All solvents were commercially available grade. All reactions were carried out under argon atmosphere, unless otherwise mentioned. Reaction mixtures were purified by flash column chromatography using silica gel high purity grade (Merck grade 9385 pore size 60 Å, 230–400 mesh particle size). Reaction mixtures were analyzed by analytical thin‐layer chromatography (TLC) using plates precoated with silica gel (Merck 60 F254, 0.25 mm). Visualization was accomplished with UV light (254 nm). ^1^H NMR spectra were recorded on a Bruker spectrometer at 400 MHz in DMSO‐_*d6*_, using tetramethylsilane (TMS) as an internal standard. Chemical shifts were reported in parts per million (ppm) on the δ scale from an internal standard (NMR descriptions: s, singlet; d, doublet; t, triplet). Mass spectroscopy was performed using an ESI‐FIA‐TOF micrOTOF spectrometer.

#### 2.1.2 Procedure for the Synthesis of the 6,7‐Diacetoxy‐3‐(2‐Nitrophenyl)Coumarin

This coumarin was synthesized under anhydrous conditions, using material previously dried at 60 °C for at least 12 h and at 300 °C for a few minutes immediately before use. A solution containing anhydrous potassium acetate (CH_3_CO_2_K, 2.94 mmol), 2‐nitrophenylacetic acid (1.67 mmol), and 2,4,5‐trihydroxybenzaldehyde (1.67 mmol), in acetic anhydride (Ac_2_O, 1.2 mL), was refluxed for 16 h. The reaction mixture was cooled, neutralized with 10% aqueous sodium bicarbonate (NaHCO_3_), and extracted with ethyl acetate (EtOAc, 3 × 30 mL). The organic layers were combined, washed with distilled water, dried with anhydrous sodium sulfate (Na_2_SO_4_), and evaporated under reduced pressure. The product was purified by recrystallization in ethanol (EtOH) and dried to afford the titled compound. Yield 52%. MS *m/z*: calculated: 383.0672; obtained: 384.0710 (M + 1).

#### 2.1.3 Procedure for the Synthesis of the 6,7‐Dihydroxy‐3‐(2‐Nitrophenyl)Coumarin

This coumarin was obtained by hydrolysis of its acetoxylated counterpart. 6,7‐Diacetoxy‐3‐(2‐nitrophenyl)coumarin, mixed with 2 N aqueous hydrochloric acid (HCl) and methanol (MeOH), was refluxed during 3 h. The resulting reaction mixture was cooled in an ice bath and the reaction product, obtained as solid, was filtered, washed with cold distilled water, and dried under vacuum to afford the titled compound. Yield = 80%. ^1^H NMR (400 MHz, DMSO‐_*d6*_) δ (ppm), *J* (Hz): 10.34 (s, 1H, OH), 9.50 (s, 1H, OH), 8.09 (s, 1H, H‐4), 8.03 (d, *J* = 7.9, 1H, H‐3′), 7.78 (t, *J* = 7.9, 1H, H‐4′), 7.62 (t, *J* = 7.9, 2H, H‐5′, H‐6′), 7.06 (s, 1H, H‐8), 6.78 (s, 1H, H‐5). MS *m/z*: calculated: 299.0430; obtained: 300.0500 (M + 1).

### 2.2 Toxicology

#### 2.2.1 Chemicals

Methyl methane sulfonate (MMS, Sigma‐Aldrich, CAS number 66–27‐3) and benzo(*a*)pyrene (BaP, Sigma‐Aldrich) were used as positive controls due to the well‐established DNA damage induced by the compounds in the comet and micronucleus (MN) assays. The chemicals used were obtained from the following suppliers: trypsin (Sigma‐Aldrich), phytohemagglutinin (Sigma‐Aldrich), cytochalasin‐B (Sigma‐Aldrich), normal melting point (NMP) agarose (Invitrogen), low melting point (LMP) agarose (Invitrogen), fetal bovine serum (FBS, Gibco), ethylenediaminetetraacetic acid (EDTA, Merck), Triton X‐100 (JTBaker), resazurin (Sigma‐Aldrich), ethidium bromide (Sigma‐Aldrich), Giemsa (Synth), and histopaque‐1077 (Sigma‐Aldrich).

#### 2.2.2 Cell Types

Peripheral blood mononuclear cells (PBMCs), isolated from peripheral blood, were obtained by venipuncture from four young (18–30 years old) healthy donors (not under pharmacological treatment). Blood samples were collected with written informed consent from the donors. The Human Ethics Committee of the Universidade Estadual Paulista (UNESP), in Marília, Brazil, approved the present study by opinion no. 5,951,710, on March 9, 2023. PBMC cells were cultured in RPMI 1640 medium, 15–20% FBS, and antibiotic‐antimycotic (1%) at a temperature of 37 °C, 5% CO_2_, and 95% relative humidity. The human hepatoma cell line (HepG2/C3A)—metabolizing cells—was obtained from the Rio de Janeiro Cell Bank, located at the Federal University of Rio de Janeiro, Brazil. Cells were cultured in Dulbecco's minimum essential medium (DMEM, Gibco) with pyruvate and L‐glutamine, supplemented with 15–20% FBS (Gibco) and 1% penicillin at 37 °C, 5% CO_2_, and 95% air in a humidified incubator.

#### 2.2.3 Cytotoxicity Assay

Based on the fact this family of coumarins has biological effects at low concentrations (Ferreira et al. 2022), concentrations of 0.01, 0.1, 1, 10, and 50 μg/mL were chosen to be tested in the cytotoxicity experiments on PBMC and HepG2/C3A cells, following the resazurin assay. The test was performed according to the manufacturer's guidelines. The cells were seeded in 96‐well plates at 2.5 × 10^4^ cells/well, with 4 wells per concentration, with a total volume of 100 μL of medium supplemented with FBS. After stabilization for 24 h, the cells were subjected to treatment with the test coumarin for 24 and 48 h, with a final volume of 200 μL. In separate wells, positive and negative controls were performed to validate the experiments. The positive control used was Triton X‐100 diluted in culture medium (without FBS) at a concentration of 1%. The negative control was cells without any type of treatment and for the blank, pure culture medium (without FBS). At the end of the treatment, the culture medium containing compound and controls was removed, and the cells were subjected to 100 μL solution of resazurin diluted in DMEM without FBS at 60 μM, and incubated at 37 °C for 3 h. Plates were analyzed with a spectrophotometric microplate reader, using a 520 nm excitation filter (Epoch‐Biotek). Cell viability (expressed as a percentage) was calculated according to the following formula: cell viability (%) = (average fluorescence of the treated group − absorbance value of the blank) / (average fluorescence of the control − absorbance value of the blank) × 100.

#### 2.2.4 Comet Assay

PBMC and HepG2/C3A cells were used for performing the comet assay as described by Tice et al. (2000). Firstly, slides were prepared by adding a thin and uniform layer of normal melting point (NMP) agarose to their surface. PBMC and HepG2/C3A cells were added at a density of 2 × 10^5^ to 24‐well plates. HepG2/C3A cells were kept for 24 h for cell attachment. Treatment was made using the 6,7‐dihydroxy‐3‐(2‐nitrophenyl)coumarin at 1, 10, and 30 μg/mL. The concentrations were chosen considering cell viability superior to 75% (by the resazurin assay). MMS (45 mM) was used as a positive control and 1% DMSO solution as a negative control. The treatments occurred for 4 h and, after this time, 10 μL of the cell sample was suspended in 0.5% LMP agarose prepared in PBS, pipetted onto the frosted microscopic glass slides precoated with NMP agarose, and kept at 4 °C for 15 min for solidification. After completing this step, slides were placed in the lysis buffer (pH 10) for 2 h at 4 °C. After lysis, slides were moved to an electrophoresis chamber and covered with an alkaline buffer (300 mM NaOH and 1 mM EDTA, pH < 13). Upon 20 min resting with the alkaline buffer, electrophoresis was performed in an ice bath (4 °C) for 20 min at 25 V and 300 mA (0.722 V/cm). Slides were then transferred to a vat with a neutralization buffer (0.4 M Tris–HCl, pH 7.5) and kept for 15 min. After neutralization, slides were dried at room temperature, fixed in 100% ethyl alcohol for 10 min, dried again, and stored overnight before staining. For staining, slides were firstly cleaned with distilled water. Then, 30 μL of 1 ethidium bromide staining solution were added to each slide, and slides were then covered with coverslips. The visual scoring of comets was performed in a fluorescence microscope (Olympus, 515–560 nm excitation filter, 590 nm barrier filter, 400× magnification). All experiments were performed three times. At least 100 cells (50 cells per slide) were scored per culture well. Cells were classified according to four classes (tail size): class 0, no tail; class 1, tail shorter than the head (nucleoid) diameter; class 2, tail length 1 to 2× larger than the head diameter; class 3, tail length more than twice the head diameter. Comets having almost the whole DNA in the tail were scored separately since these probably represented dead cells (Hartmann et al. 2003; OECD 489 2016). The total score (sum of multiplying the number of cells in each class with the damage class) ranged from 0 (no damage) to 300 (severe damage).

#### 2.2.5 Cytokinesis Block Micronucleus (CBMN) Assay

CBMN assay was performed as described by Fenech (2000). The same 6,7‐dihydroxy‐3‐(2‐nitrophenyl)coumarin concentrations used in the comet assay were used in this assay. Human peripheral blood lymphocytes (PBLs) from two donors were collected (blood samples). HepG2/C3A cells were kept in a 25 cm^2^ culture flask. Experiments were carried out four times for PBL and in triplicate for HepG2/C3A cells. MMS (150 mM) was used as a positive control for PBL and B(a)P (2 mM) for HepG2/C3A cells. 1% DMSO solution was used as a negative control. To the PBL cultures, each 10 cm^2^ flat face culture tube (TPP) was prepared with 5 mL supplemented RPMI 1640 medium, adding 5 μg/mL of phytohemagglutinin‐A to stimulate lymphocyte division and 0.5 mL plasma, followed by 6 to 8 drops erythrocyte concentrates. The tubes were incubated for 72 h at 37 °C and 5% CO_2_. The exposure to the three different concentrations of 6,7‐dihydroxy‐3‐(2‐nitrophenyl)coumarin was done 44 h later. Four hours after the addition of the test compound, cytochalasin‐B (6 μg/mL) was added to each culture until 72 h of incubation. For HepG2/C3A cells, 25 cm^2^ culture flasks (TPP) were prepared with 5 mL supplemented DMEM high glucose at a cell concentration of 2 × 10^5^. The cells were cultured at 37 °C and 5% CO_2_ until a complete cell cycle (24 h). Then, the exposure to the three concentrations of 6,7‐dihydroxy‐3‐(2‐nitrophenyl)coumarin was made for 24 h. B(a)P (2 μM) was employed as a positive control (OECD TG 487 2023). The negative control was 1% DMSO. After this time, the culture medium of all flasks was discarded, and the cells were washed with 5 mL PBS, which was removed. Cytochalasin B (6 μg/mL) was added to each flask together with culture medium, and the flasks were incubated for 28 h more. Next, the cells were washed and transferred to centrifuge tubes after treatment with trypsin 0.5% EDTA. After the above steps, the cultures of both cell types were centrifuged for 5 min, and the pellets were suspended in 0.075 M KCl hypotonic solution and 10 mg/mL sodium citrate solution, both at 4 °C, respectively, for lymphocytes and HepG2/C3A cells. Again, the cells were centrifuged and fixed in a cold solution of a 3:4 and 3:1 ratio of methanol and acetic acid for lymphocytes and HepG2/C3A, respectively. This step was repeated twice, and a 5:1 ratio fixative for lymphocytes was used. In the first fixation, 4% formaldehyde drops were added for cytoplasm preservation. The slides were prepared by dripping and stained with 10% Giemsa for 10 min. The analysis was performed in the light microscope (Zeiss, Primo Star) with 400× magnification, with the count of 1000 binucleated cells for exposure/repetition and controls, noting the presence of micronuclei, nucleoplasmic bridges, and nuclear buds in the cells. As a measure of cytotoxicity, the cytokinesis‐block proliferation index (CBPI) was calculated, following the formula: CBPI = (𝑀1 + 2𝑀2 + 3𝑀3)/𝑁, where 𝑀1‐𝑀2 represents the number of cells with 1–2 nuclei, 𝑀3 represents the number of cells with more than 2 nuclei, and 𝑁 (500) is the total number of scored cells (OECD 487 2023).

### 2.3 Statistical Analysis

After checking for normal distribution, statistical analysis was performed with analysis of variance (ANOVA) followed by Dunnett's and/or Tukey's post‐test using GraphPad Prism 5 software. The results were considered statistically significant at *p* < 0.05.

## 3 Results

### 3.1 Chemistry

**FIGURE 1:**
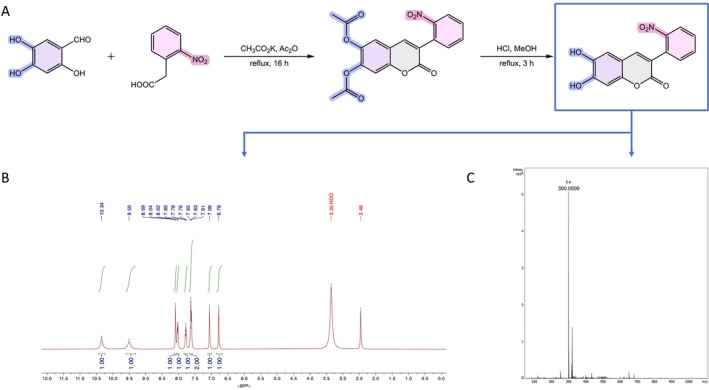
(A) Synthetic methodology and reaction conditions for the synthesis of 6,7‐dihydroxy‐3‐(2‐nitrophenyl)coumarin. (B) ^1^H NMR spectrum. (C) ESI mass spectrum.

6,7‐Dihydroxy‐3‐(2‐nitrophenyl)coumarin has been obtained via the Perkin–Oglialoro reaction, following the conditions represented in Figure 1A. This procedure occurs in two different steps: (1) the formation of the coumarin ring with acetylation of the hydroxyl groups and (2) acidic deacetylation to afford the correspondent hydroxyl derivative. In summary, as described in the materials and methods section, all components (2‐nitrophenylacetic acid, 2,4,5‐trihydroxybenzaldehyde, potassium acetate, acetic anhydride) stir in a round‐bottom flask for 16 h at reflux temperature. Under these conditions, both the acetylation of the hydroxyl groups and the closure of the pyrone ring occur simultaneously. Afterwards, the 6,7‐diacetoxy‐3‐(2‐nitrophenyl)coumarin is subjected to deacetylation in the presence of aqueous hydrochloric acid (HCl) and methanol (MeOH), at reflux temperature, for 3 h. The final product was obtained in 80% yield and then characterized by proton nuclear magnetic resonance (^1^H NMR, Figure 1B) and mass spectrometry (Figure 1C). The presence of the H‐4 peak in the ^1^H NMR spectrum around 8 ppm, along with the methyl peak integrating for 6 protons from the acetoxy groups at 2.36 ppm, confirms the first step of the reaction. The disappearance of the methyl groups peak at 2.36 ppm, accompanied by the appearance of two new peaks corresponding to the hydroxyl groups at 9.50 and 10.34 ppm, confirms the second step of the reaction (Figure 1B).

### 3.2 Cell Viability Test

**FIGURE 2:**
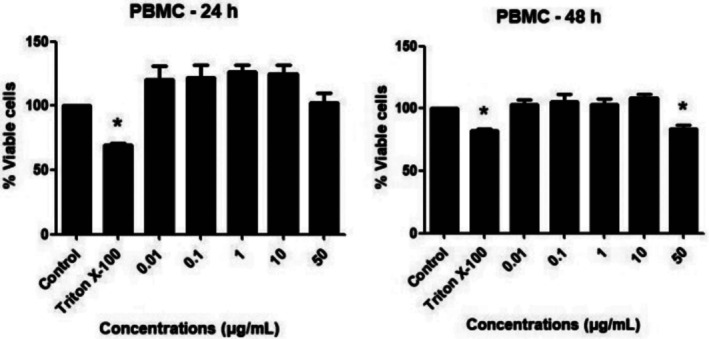
Percentage of viable PBMC cells after 24 and 48 h of exposure to different concentrations of 6,7‐dihydroxy‐3‐(2‐nitrophenyl)coumarin (μg/mL), evaluated by the viability test with resazurin. Significantly different from the negative control: *p < 0.05.

**FIGURE 3:**
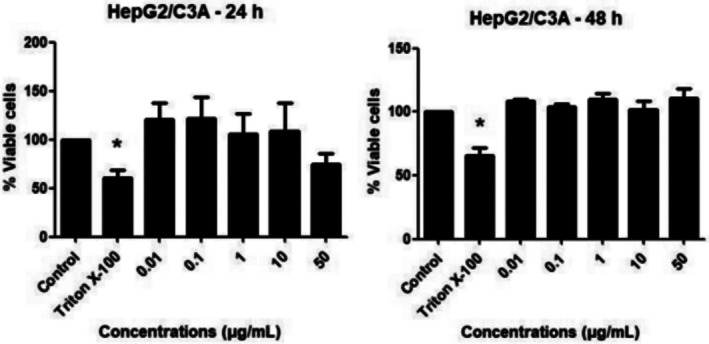
Percentage of viable HepG2/C3A cells after 24 and 48 h of exposure to different concentrations of 6,7‐dihydroxy‐3‐(2‐nitrophenyl)coumarin (μg/mL), evaluated by the viability test with resazurin. Significantly different from the negative control: *p < 0.05.

Figure 2 shows cell viability results using data obtained by the resazurin test for PBMC cells treated with different concentrations of 6,7‐dihydroxy‐3‐(2‐nitrophenyl)coumarin. All concentrations tested showed cell viability ≥ 75% after 24 and 48 h of exposure. Regarding HepG2/C3A, up to the concentration of 10 μg/mL, no decrease in cell viability was observed. However, at the highest concentration tested of 50 μg/mL, at the shortest exposure time (24 h) there was a small and significant reduction, as observed in Figure 3. As expected, for both cell types, 1% Triton X‐100, used as a positive control, resulted in a significant reduction in cell viability.

### 3.3 Genotoxicity Tests

Data on the comet assay, with the total number of damaged cells and scores for PBMC and HepG2/C3A cells exposed to concentrations of 1, 10, and 30 μg/mL of 6,7‐dihydroxy‐3‐(2‐nitrophenyl)coumarin, are presented in Tables 1 and 2, respectively. As expected, MMS—used as a positive control—induced a significant increase in DNA damage, with migration of DNA pieces to the tail, in both cell types. In the cells treated with the different concentrations of the synthetic coumarin under study, after 4 h of exposure, within the experimental conditions used here, no significant DNA damage was observed. In the few cells in which DNA damage was observed, this was class 1 (little damage), also observed for the negative control.

**TABLE 1:** DNA damage assessed by comet assay of 6,7‐dihydroxy‐3‐(2‐nitrophenyl)coumarin in PBMC cells, after 4 h of treatment.

Comet assay						
Treatments	Total^a^	0	1	2	3	Scores
**Control**	20.25 ± 6.133	79.75 ± 6.1	14.50 ± 5.50	4.50 ± 1.73	1.25 ± 0.50	27.25 ± 6.85
**Coumarin (1 μg/mL)**	3.50 ± 1.91	96.50 ± 1.91	2.50 ± 1.73	1.00 ± 0.81	0.00 ± 0.00	4.50 ± 2.38
**Coumarin (10 μg/mL)**	5.00 ± 0.81	95.25 ± 1.00	3.25 ± 0.57	1.25 ± 0.57	0.50 ± 0.57	7.25 ± 1.70
**Coumarin (30 μg/mL)**	7.50 ± 3.87	92.50 ± 9.87	6.00 ± 4.08	0.50 ± 1.00	1.00 ± 0.81	10.00 ± 4.54
**MMS (45 μM)**	84.00 ± 8.86*	16.00 ± 8.86*	44.75 ± 3.86*	30.75 ± 6.65*	8.50 ± 3.10*	131.80 ± 20.66*

*Note:* DNA damage was expressed as “arbitrary units.” Control = DMSO 1%. Mean ± standard deviation of four replicates.

* Significantly different from negative control (*p* < 0.05), one‐way analysis of variance and Dunnett's test.

^a^ Total number of damaged cells (class 1 + 2 + 3).

**TABLE 2:** DNA damage assessed by comet assay of 6,7‐dihydroxy‐3‐(2‐nitrophenyl)coumarin in HepG2/C3A cells, after 4 h of treatment.

Comet assay						
Treatments	Total^a^	0	1	2	3	Scores
**Control**	9.33 ± 0.57	92.33 ± 1.15	4.33 ± 1.15	0.66 ± 0.57	4.33 ± 1.52	18.67 ± 3.78
**Coumarin (1 μg/mL)**	7.66 ± 1.52	92.33 ± 1.52	4.33 ± 0.57	1.33 ± 1.15	2.00 ± 0.00	13.00 ± 2.64
**Coumarin (10 μg/mL)**	7.00 ± 2.64	93.00 ± 2.64	4.66 ± 1.52	0.33 ± 0.57	2.00 ± 1.00	11.33 ± 5.03
**Coumarin (30 μg/mL)**	6.33 ± 0.57	93.66 ± 0.57	4.33 ± 0.57	0.33 ± 0.57	1.66 ± 0.57	10.00 ± 1.00
**MMS (45 μM)**	86.00 ± 3.00*	14.66 ± 2.08*	28.66 ± 2.08*	45.00 ± 2.64*	12.33 ± 2.30*	155.7 ± 6.50*

*Note:* DNA damage was expressed as “arbitrary units.” Control = DMSO 1%. Mean ± standard deviation of four replicates.

* Significantly different from negative control (*p* < 0.01), one‐way analysis of variance and Dunnett's test.

^a^ Total number of damaged cells (class 1 + 2 + 3).

Regarding the CBMN test, the results of the analysis of the clastogenic/aneugenic potential of the 6,7‐dihydroxy‐3‐(2‐nitrophenyl)coumarin are presented in Tables 3 and 4. It was observed that in both cell types treated with the compound under study, there were no significant differences in the frequencies of binucleated cells with micronuclei, nucleoplasmic bridges, and nuclear buds in relation to the negative control. As expected, both substances used as positive controls—MMS and B(a)P—presented a significant clastogenic/aneugenic effect in the cells, attesting to the sensitivity of the assay in the detection of chromosomal mutations.

**TABLE 3:** Frequency of micronuclei (MN), nucleoplasmic bridge (PN), nuclear buds (BN) and cytokinesis block proliferation index (CBPI) in PBMC cells exposed to different concentrations of 6,7‐dihydroxy‐3‐(2‐nitrophenyl)coumarin.

	Binucleated cells					
		MN (4000 cells)		PN No.	NB No.	CBPI (500 cells) (Mean ± SD)
Tested compound	Concentration (μg/mL)	No.	%			
**Negative control**	0	6	0.15	7	0	1.308 ± 0.10
**Positive control**	150^a^	44*	1.1*	22*	8*	1328 ± 0.08
**Coumarin**	1	16	0.4	14	6	1.313 ± 0.07
	10	13	0.325	15	1	1.388 ± 0.03
	30	12	0.3	7	2	1.339 ± 0.10

*Note:* Negative control: RPMI. Positive control: MMS.

Abbreviations: BN, nuclear bud; MN, micronucleus; PN, nucleoplasmic bridge; SD, standard deviation.

* Statistically different from the negative control (*p* < 0.05), one‐way analysis of variance (ANOVA) and Dunnett test.

^a^ Two micromolar.

**TABLE 4:** Frequency of micronuclei (MN), nucleoplasmic bridge (PN), nuclear buds (BN) and cytokinesis block proliferation index (CBPI) in HepG2/C3A cells exposed to different concentrations of 6,7‐dihydroxy‐3‐(2‐nitrophenyl)coumarin.

	Binucleated cells					
		MN (3000 cells)		PN No.	NB No.	CBPI (500 cells) (Mean ± SD)
Tested compound	Concentration (μg/mL)	No.	%			
**Negative control**	0	7	0.233	3	2	1.359 ± 0.08
**Positive control**	2^a^	29*	0.97*	9*	6*	1.373 ± 0.01
**Coumarin**	1	13	0.43	0	2	1.322 ± 0.02
	10	12	0.4	1	2	1.347 ± 0.04
	30	7	0.233	4	0	1.282 ± 0.05

*Note:* Negative control: RPMI. Positive control: MMS.

Abbreviations: BN, nuclear bud; MN, micronucleus; PN, nucleoplasmic bridge; SD, standard deviation.

* Statistically different from the negative control (*p* < 0.05), one‐way analysis of variance (ANOVA) and Dunnett test.

^a^ Two micromolar.

## 4 Discussion

The cytotoxic and genotoxic potential of the synthetic 6,7‐dihydroxy‐3‐(2‐nitrophenyl)coumarin was investigated for the first time in human cells in culture. The first aspect investigated in the present study was the cytotoxic potential of the molecule through the resazurin assay, using metabolically non‐competent cells, PBMC, and human hepatoma HepG2/C3A cells, proficient in this metabolization. In this assay, viable cells are able to reduce resazurin, which has a blue coloration, into resorufin, which has a pink coloration (Niles et al. 2013). The color change can be detected and quantified by spectrophotometer and the data obtained transformed into a percentage of viable cells. The cytotoxicity data obtained from the test showed that up to a concentration of 10 μg/mL, no reduction in viability was observed in both cell types after 24 and 48 h of exposure. However, in PBMC cells at a concentration of 50 μg/mL, for the shortest exposure time, a significant reduction in the percentage of viable cells was observed, which did not occur for the longest exposure time.

These cellular toxicity results are generally in line with other studies of synthetic coumarins already described. Fedato and Maistro (2014) and Marques et al. (2015) reported the absence of cytotoxicity of the 4‐methylesculetin and esculetin, two well‐known simple coumarins, in different cell lines. Some natural coumarins, which have high trypanocidal activity, were tested in vitro in human lymphocytes using a similar cytotoxicity assay, the MTT test, and also showed no cytotoxic effects (Fedato and Maistro 2014; Marques et al. 2015; Reyes‐Chilpa et al. 2008). In another study, Salar et al. (2019) reported the absence of cytotoxicity of 13 biscoumarins with antiglycant and urease inhibitory properties, using the same MTT metabolization viability test. More recently, Castanha et al. (2023) reported that 3‐(3,4‐dihydroxyphenyl)‐7,8‐dihydroxycoumarin does not produce cytotoxic effects in HepG2/C3A cells and PBMC after 24, 48, and 72 h of exposure. On the other hand, studies by Pereira et al. (2023) described that 3‐(3‐hydroxyphenyl)‐7‐hydroxycoumarin, at the highest concentrations tested and at the longest exposure times (48 and 72 h), promoted a significant reduction in the cell viability of PBMC cells, similarly to the observed results described herein. In contrast to the data reported above, Silva et al. (2023) reported that 3‐(3,4‐dihydroxyphenyl)‐8‐hydroxycoumarin, under the same experimental conditions, produced a significant reduction in viability in HepG2/C3A cells after 48 and 72 h of exposure to concentrations between 0.1 and 20 μg/mL. The difference between these two studied compounds is only the presence of a hydroxyl at position 7 of the scaffold, which leads to significant differences in the cytotoxic potential (Silva et al. 2023). In general, the data indicate that the chemical structure of the coumarins, the concentration, and the time of exposure are important factors in determining the cytogenotoxic response against different cell types.

Another parameter investigated in this study was the potential of 6,7‐dihydroxy‐3‐(2‐nitrophenyl)coumarin to cause damage to the DNA, using the comet assay. This assay, also known as single‐cell gel electrophoresis, allows the detection of breaks in both single and double strands of DNA, at the level of individual cells. The biological consequence of this type of DNA damage, if not repaired by the cell, is the formation of mutations, development of cancer, genetic diseases, and even cell death (Collins et al. 2008; Møller et al. 2020). The data obtained in the assay showed that 6,7‐dihydroxy‐3‐(2‐nitrophenyl)coumarin does not cause DNA damage in cells with and without the capacity to metabolize xenobiotics. For other hydroxycoumarins studied by our research group, the genotoxic potential varied according to the position of the hydroxyl in the molecule. In the study by Castanha et al. (2023), DNA damage was not observed after exposure of leukocytes and HepG2/C3A cells to coumarin 3‐(3,4‐dihydroxyphenyl)‐7,8‐dihydroxycoumarin, up to the maximum tested concentration (10 μg/mL). The same was observed in the study by Pereira et al. (2023), for the 3‐(3‐hydroxyphenyl)‐7‐hydroxycoumarin. In the study by Silva et al. (2023), for the 3‐(3, 4‐dihydroxyphenyl)‐8‐hydroxycoumarin, up to a concentration of 10 μg/mL, any genotoxicity signs were registered (Silva et al. 2023). However, when studying a higher concentration of 20 μg/mL, significant damage to the DNA of PBMC cells was observed. Once again, a pattern of cellular and/or genetic toxicity of synthetic hydroxyphenylcoumarins on the genome of human cells can be observed as the exposure and concentrations increase. In comparison with these three coumarins already studied by the research group, the one investigated in the current study was tested up to a higher concentration of 30 μg/mL, and at this concentration, genotoxic effects were not observed.

The third and final toxicity parameter investigated in this study was the ability of the tested coumarin to cause chromosomal mutations in human cells with and without hepatic metabolization capacity. The micronucleus test is used to evaluate chromosomal breaks and changes in chromosome number. Micronuclei are formed during anaphase in the cell cycle, where daughter chromosomes move to opposite ends of the cell. Entire chromosomes that are delayed in moving or chromosome fragments, induced by DNA damage, form these micronuclei, which in the telophase phase may or may not be included in the nucleus of the daughter cells (Fenech, 2000; OECD 487 2023). Using the micronucleus test with cytokinesis blockade, data from the analysis of the clastogenic/aneugenic potential of 6,7‐dihydroxy‐3‐(2‐nitrophenyl)coumarin also show that none of the concentrations tested caused chromosomal alterations in both cell types. Performing the same assay, similar concentrations, and the same cell types, Castanha et al. (2023), analyzing 3‐(3,4‐dihydroxyphenyl)‐7,8‐dihydroxycoumarin, Silva et al. (2023), investigating 3‐(3,4‐dihydroxyphenyl)‐8‐hydroxycoumarin, and Pereira et al. (2023), studying the potential mutagenicity of 3‐(3‐hydroxyphenyl)‐7‐hydroxycoumarin, did not observe the occurrence of any chromosomal mutations. Considering that a the synthetic hydroxycoumarins that have been studied present their pharmacological activity at very low concentrations, in the nano or low micromolar ranges, associated with a half‐life of less than 24 h (Matos et al. 2015), the potential toxicity of these coumarins, which have been slightly observed at high concentrations and longer exposure times, may not be therapeutically relevant.

An important point to consider is that, although the HepG2/C3A cell line is widely used and recommended in xenobiotic cytotoxicity and genotoxicity tests, these cells do not cover all possible hepatic metabolic pathways (Knasmüller et al. 1998). Therefore, the use of other forms of metabolism, such as the use of the S9 fraction from rodents, would be an important aspect to investigate in future studies of these coumarins.

## 5 Conclusion

In conclusion, the studied compound demonstrated a lack of toxicity and genotoxicity at therapeutic concentrations on cells with active hepatic metabolism (HepG2/C3A), as well as non‐metabolizing cells (PBMC), highlighting its safety profile. The cytogenotoxicity data support the absence of cellular toxicity at lower concentrations, which are within the range of potential therapeutic doses, as observed for previously studied 3‐arylcoumarins. These promising results provide a strong foundation for continued research and development, reinforcing the potential of these compounds as future drugs or nutraceuticals for human use.

## Author Contributions

**Julia Zocca Nunes de Barros**: methodology, data collection, writing original. **Luma Eduarda Lopes Escalante:** methodology, data collection. **Maria João Matos:** methodology, conceptualization, resources, writing review. **Edson Luis Maistro**: conceptualization, supervision, funding acquisition, writing original – review and editing.

## Conflicts of Interest

The authors declare no conflicts of interest.

## Data Availability

Data available on request from the authors.
